# Effects of Soy Protein Isolate and Inulin Conjugate on Gel Properties and Molecular Conformation of Spanish Mackerel Myofibrillar Protein

**DOI:** 10.3390/foods13182920

**Published:** 2024-09-15

**Authors:** Wei Wang, Sirui Ma, Qing Shao, Shumin Yi

**Affiliations:** 1 College of Food Science and Engineering, Bohai University, Jinzhou 121013, China; wwll812002@163.com (W.W.); masirui0228@163.com (S.M.); shaoqing1939827103@163.com (Q.S.); 2 National & Local Joint Engineering Research Center of Storage, Processing and Safety Control Technology for Fresh Agricultural and Aquatic Products, National R&D Branch Center of Surimi and Surimi Products Processing, Jinzhou 121013, China

**Keywords:** soy protein isolate, inulin, myofibrillar protein, secondary structure, gel properties

## Abstract

The gel properties and molecular conformation of Spanish mackerel myofibrillar protein (MP) induced by soy protein isolate–inulin conjugates (SPI–inulin conjugates) were investigated. The addition of SPI–inulin conjugates significantly enhanced the quality of the protein gel. An analysis of different additives was conducted to assess their impact on the gel strength, texture, water-holding capacity (WHC), water distribution, intermolecular force, dynamic rheology, Raman spectrum, fluorescence spectrum, and microstructure of MP. The results demonstrated a substantial improvement in the strength and water retention of the MP gel with the addition of the conjugate. Compared with the control group (MP), the gel strength increased from 35.18 g·cm to 41.90 g·cm, and WHC increased from 36.80% to 52.67% with the inclusion of SPI–inulin conjugates. The hydrogen bond content was notably higher than that of other groups, and hydrophobic interaction increased from 29.30% to 36.85% with the addition of SPI–inulin conjugates. Furthermore, the addition of the conjugate altered the secondary structure of the myofibrillar gel, with a decrease in α-helix content from 62.91% to 48.42% and an increase in β-sheet content from 13.40% to 24.65%. Additionally, the SPI–inulin conjugates led to a significant reduction in the endogenous fluorescence intensity of MP. Atomic force microscopy (AFM) results revealed a substantial increase in the Rq value from 8.21 nm to 20.21 nm. Adding SPI and inulin in the form of conjugates is an effective method to improve the gel properties of proteins, which provides important guidance for the study of adding conjugates to surimi products. It has potential application prospects in commercial surimi products.

## 1. Introduction

Surimi serves as a stable myofibrillar protein (MP) concentrate derived from boneless fish through rinsing and dehydration, serving as an intermediate raw material for the production of various value-added products [1]. According to the statistics of the China Fishery Statistical Yearbook in 2023, the total output of aquatic products in 2022 will reach 68.66 million tons, an increase of 2.62% over 2021, of which the amount of marine fish and freshwater fish farming will reach 53.94 million tons, and surimi products will reach 1.35 million tons, an increase of 0.37% over 2021. Spanish mackerel (*Scomberomorus niphonius*), renowned for its delectable taste, nutritional richness, and dense meat, has emerged as a crucial raw material for surimi and surimi product manufacturing [1].

To reduce costs, improve gel quality, and improve the nutritional value of products, practitioners and researchers usually add exogenous additives to surimi and change processing methods in the actual production of surimi products and the development of new products. MP, a salt-soluble protein, primarily comprises Myosin, Actin, Troponin-T, and Tropomyosin [1,2], constituting approximately 50% of the total protein content. As the principal protein in fish meat, MP plays a vital role in surimi composition, influencing the gel properties crucial for the sensory evaluation of surimi products [3]. In the process of protein gelation, the MP gel network unfolds, aggregates, and then forms, finally forming an elastic surimi gel with a three-dimensional network structure [4]. By studying the changes in myofibrillar protein, the effects of exogenous additives or processing methods on surimi gel were analyzed. Ultrasonic cavitation proves influential in inducing the conformational unfolding of muscle proteins, exposing more active sites, and facilitating robust protein interactions. This effect contributes to the formation of high-quality elastic gels [5,6]. Given the close relationship between MP gel properties and meat product gel quality, enhancing the gel properties of MP holds paramount importance in meat production.

Exogenous additives, in particular, have been leveraged to alter protein conformation and enhance the gel properties of meat products. For example, adding insoluble dietary fiber to improve taste, gel quality, and nutritional value has been explored [7]. Additionally, the RC emulsion was obtained by acid-dissolved and water-regenerated microcrystalline cellulose, and the addition of 30% improved the rheology, structural properties, and microstructure of MP gel, filled the network structure of MP, therefore improving the quality of meat products [8]. Sugarcane dietary fiber, when added to MP, promotes α-helix expansion and β-sheet formation, enhancing gel strength [7]. High-pressure synergistic heat treatments have been employed to transform the α-helix of MP into a β-sheet, elevating its gel-forming ability [9]. Notably, the incorporation of 3% potato dietary fiber with chicken MP demonstrated a significant reduction in α-helix content [10]. Additionally, soy protein isolate (SPI), after acid micro-heat treatment, was found to interact with myosin heavy chain and actin, promoting protein gel formation [11]. Therefore, strengthening research on the conformational changes and gel properties of MP was crucial for advancing surimi product production practices.

SPI stands out as the primary product derived from defatted soybean meal, serving as a crucial source of plant protein in food production and a significant contributor to human protein intake [12]. Due to its high nutritional value and commendable functional characteristics, SPI holds vast potential for applications in various foods [12]. According to different sedimentation coefficients, SPI can be categorized into 2S, 7S, 11S, and 15S, among which glycinin (11S) and β-conglycinin (7S) constitute approximately 70% of the total protein content [13]. The 7S class comprises trimeric glycoproteins composed of three subunits: α (molecular weight: approximately 71 KDa), α′ (molecular weight: approximately 67 KDa), and β (molecular weight: approximately 50 KDa) [14]. On the other hand, 11S consists of an acidic polypeptide A (molecular weight: approximately 35 KDa) and a basic polypeptide B (molecular weight: approximately 20 KDa) [15]. However, SPI prepared through conventional acid-base precipitation separation methods exhibits poor solubility and emulsification, limiting its application in the food industry [16]. The Maillard reaction offers a means to modify the rigid structure of SPI, expanding its potential applications in the food industry [14]. Studies have shown that SPI can enhance the gel properties of MP, dependent on formulation conditions and soy protein properties [17]. For instance, AH-SPI interacts effectively with muscle proteins, promoting the water-holding capacity (WHC) of MP gel and reducing relaxation time. The addition of AH-SPI was conducive to the formation of MP gels with a more continuous and uniform gel network structure [18]. Notably, there was evidence that natural SPI does not interact well with meat protein, negatively affecting the elasticity and hardness of MP gels [19]. Preheating has been found to enhance the gelling properties of soy proteins by moderately denaturing the proteins, making them more amenable to cross-linking [20]. Therefore, modifying the spatial structure of natural SPI becomes essential to enhance its applicability, and this can be achieved through structural modifications.

Inulin is a carbohydrate composed of β-(2,1)-linked fructose units, mostly ending with a glucose unit. As a naturally soluble dietary fiber, inulin can control blood lipids, improve intestinal function, and promote mineral absorption and other functions, it can also enhance the satiety of the human body [21]. For example, by combining pea protein isolate with inulin through pH shift and ultrasonic assistance, the prepared complex has good thermal stability and oxidation resistance and significantly improves the oxidation stability and tolerance of algal oil emulsion [22].

While existing research has primarily focused on the impact of SPI or inulin on MP gel properties, the potential mechanisms and molecular structures underlying the effects of SPI–inulin conjugates remain unexplored. This study introduces SPI–inulin conjugates to MP and employs various analyses, including gel strength, texture profile analysis (TPA), WHC, water distribution, intermolecular force, secondary structure, rheological properties, endogenous fluorescence spectrum, microstructure, and correlation analysis to elucidate its gel properties and molecular conformation. We herein aimed to comprehend the interaction between SPI–inulin conjugates and MP and provide a detailed explanation of the mechanism by which SPI–inulin conjugates influence the gel properties and molecular conformation of MP. This research was anticipated to offer crucial guidance for studies on the addition of conjugates in surimi products, ultimately enhancing the quality of surimi products.

## 2. Materials and Methods

### 2.1. Materials

Spanish mackerel, averaging 40 ± 4 cm in length and weighing 1000 ± 50 g, were procured from Jinzhou Keji Road Aquatic Products Market (Liaoning, China). SPI (purity 90%) was obtained from Shandong Linfen Shansong Biological Products Co., Ltd. (Shandong, China). Inulin (purity, 95%; polymerization degree, 10–12) was purchased from Hebei Fengning Ping’an Hi-tech Industrial Co., Ltd. (Hebei, China). Sodium dihydrogen phosphate and sodium dihydrogen phosphate, analytically pure, purchased from Sinopharm Group Chemical Reagent Co., Ltd. (Shanghai, China). Urea, analytical pure, purchased from Sinopharm Group Chemical Reagent Co., Ltd. (Shanghai, China). Tris, glycinate, EDTA, and DTNB, all analytically pure, were acquired from Beijing Solebao Biotechnology Co., Ltd. (Beijing, China).

### 2.2. Preparation of MP

The method of Xia et al. (2009) [23] was used to prepare MP following with slight modifications. Spanish mackerel back muscle was weighed, ground, and homogenized with four times the volume (*w/v*) of phosphate buffer (pH 7.5, 0.02 M) using a homogenizer (FJ200-SH, Shanghai Specimen Model Factory, Shanghai, China) for 2 min, and then centrifuged (LYNX4000 Biofuge Stratos, Thermo, Waltham, MA, USA) to obtain the sediment, and this process was repeated twice. The resulting precipitation was dissolved and homogenized with four times the volume (*w/v*) of NaCl (0.1 mM), and the sediment was obtained by centrifugation. Finally, the sediment was homogenized with four times the volume (*w/v*) of phosphate buffer (pH 7.5, 0.02 M) for 2 min, and the precipitation was obtained by centrifugation. The concentration of MP was determined using the Biuret method. The entire process was conducted at 4 °C with homogenization parameters set at 7500 rpm and centrifugation parameters at 8000 rpm for 20 min.

### 2.3. Preparation of SPI–Inulin Conjugates

SPI and inulin were dissolved in distilled water at a mass ratio of 1:2, and the protein concentration was adjusted to 15 mg mL^−1^. The protein was uniformly dispersed by magnetic stirring for 1 h. The sample was then subjected to an ultrasound instrument (JRA-20CQ, Wuxi Jieruian Instrument Equipment Co., Ltd., Wuxi, China), and the reactant’s temperature was controlled by heating in a water bath using a thermometer. The ultrasonic parameters were set at 82 °C, 40 min, and 300 W.

### 2.4. Preparation of Mixed MP and Gel

The concentration of MP was adjusted to 100 mg mL^−1^ with phosphate buffer (pH 7.5, 20 mmol/L). Subsequently, 0.6% SPI (MP-S), 0.6% inulin (MP-I), 0.6% mixtures of SPI and inulin (MP-M), and 0.6% SPI–inulin conjugates (MP-C) were separately added to MP. A sample with no added MP served as the control and was labeled as MP. These mixtures were then placed in gel vials and subjected to a water bath (40 °C, 30 min; 90 °C, 20 min) to form mixed myofibrillar gels. The resulting gels were chilled with ice water for 30 min and stored in a refrigerator at 4 °C overnight for subsequent analysis.

### 2.5. Gel Properties

#### 2.5.1. Determination of Gel Strength

The gel strength of each sample (height 25 mm, diameter 25 mm) was measured using a texture meter (TA-XT Plus, SMS, Godalming, UK). Experimental parameters included pre-test speed, test speed, and post-test speed, all set at 1 mm s^−1^, compression distance at 15 mm, and trigger force at 15 g. To ensure experimental accuracy, six repeated measurements were conducted for each sample.

#### 2.5.2. Determination of TPA

The TPA of the samples (height 25 mm, diameter 25 mm) was assessed using a texture meter. Test conditions comprised pre-test speed, test speed, and post-test speed set at 1 mm s^−1^, shape variable of 40%, and a trigger force of 10 g. Six repeated measurements were performed for each sample to enhance experimental reliability.

#### 2.5.3. Determination of WHC

The method described by Xu et al. was used to determine WHC, with some modifications [24]. Gel samples were sliced into approximately 3 mm thin sections, and the initial weight (W1) was measured. Subsequently, the samples were wrapped in three layers of filter paper, placed in a centrifuge tube, and centrifuged for 15 min (4 °C, 1677× *g*). After centrifugation, the final weight (W_2_) was measured, and the WHC was calculated using the formula:

(1) $$\mathrm{WHC}\left(\%\right)=\left(W2/W1\right)\;\times \;100$$

#### 2.5.4. Determination of Low-Field Nuclear Magnetic Resonance (LF-NMR)

The spin-spin relaxation time (T_2_) of the samples was assessed using a nuclear magnetic resonance (NMR) analyzer (NMI20, Newmai Analytical Instrument Co., Ltd., Suzhou, China). Briefly, the sample was placed in the NMR tube with an NMR sampler, and the relaxation time T_2_ was determined through the Carr-Purcell-Meibom-Gill (CPMG) method of the NMR analyzer. Test parameters included a proton resonance frequency of 22 MHz, repeated scanning eight times, a τ of 150 us, and 8000 echo data.

#### 2.5.5. Determination of Chemical Forces

The method described by Lu et al. [25] was used to determine chemical forces with some adjustments. Specifically, 1 g of the mixed gel sample was accurately weighed and mixed with 9 mL of 0.05 mol L^−1^ NaCl (SA), 0.6 mol L^−1^ NaCl (SB), 0.6 mol L^−1^ NaCl + 1.5 mol L^−1^ urea (SC), respectively. Then, 0.6 mol L^−1^ NaCl + 8 mol L^−1^ urea (SD) was mixed, homogenized (1341.6× *g*, 3 min), and centrifuged (2683.2× *g*, 10 min). The protein concentration in the supernatant was determined using the Coomassie brilliant blue method. The content of hydrogen bond (SC-SB), ionic bond (SB-SA), and hydrophobic interaction (SD-SC) in the gel was estimated by measuring the difference in protein content in the four solutions.

#### 2.5.6. Determination of Raman Spectrum

The method outlined by Han et al. [26] was used for Raman spectroscopy of mixed gels, followed by slight modifications. The gel samples, cut into 2 mm × 2 mm × 1 mm pieces, were placed on a clean glass slide and analyzed using a high-resolution Raman spectrometer (LabRAM HR Evolution, Digangchang China Trading Co., Ltd., Shanghai, China) equipped with a semiconductor laser (λ = 532 nm) as the excitation source. The Raman spectra of the mixed gel were measured in the wavelength range of 400–2000 cm^−1^; the contents of α-helix, β-sheet, β-turn, and random coil were calculated.

#### 2.5.7. Determination of Viscoelasticity

The rheological properties of the samples were measured using a rheometer (Discovery DHR-1, TA Company, Boston, MA, USA). The surimi was placed in parallel plates, and the storage modulus (G′) and the loss modulus (G″) were monitored using the temperature-scanning program of the rheometer. Test conditions included a temperature range of 20–90 °C, a temperature rise rate of 2 °C/min, a strain of 1%, a frequency of 0.1 Hz, and a holding time of 1 min. A 35 mm parallel steel plate geometry with a 1 mm gap was used.

### 2.6. Determination of Endogenous Fluorescence Spectrum

The method described by Han et al. [26] was used for the determination of endogenous fluorescence spectra. The MP concentration was diluted to 0.1 mg mL^−1^. Following thorough stirring, a fluorescence spectrophotometer (970CRT, Precision Scientific Instruments Co., Ltd., Shanghai, China) was employed for determination. Test parameters included an excitation wavelength of 295 nm, an emission wavelength of 310–410 nm, excitation and emission slit widths of 10 nm, and a scanning speed of 1200 nm/min.

### 2.7. Determination of Microstructure

Sample pretreatment: the MP solution was diluted to 20 μg mL^−1^. Subsequently, 10 μL of the protein solution was accurately absorbed and dropped onto a round mica sheet with a 1 cm diameter. The solution was allowed to dry naturally on a super-clean table to form a protein adsorption layer. The adsorption layer was then washed with ultra-pure water to eliminate salt interference, and the test sample was allowed to continue drying.

Atomic force microscopy (AFM) imaging: the AFM imaging utilized a scanning range of 3 μm × 3 μm for all images. Image analysis was conducted using NanoScope Run-on software v8.15.

### 2.8. Statistical Analysis

All experimental data were presented as mean ± standard deviation, and each index test was repeated at least three times. SPSS 26.0 (IBM Statistical Software^TM^, IBM Corp. Armonk, NY, USA) software was employed for the analysis of variance, and significance was determined with *p* < 0.05 according to the Duncan test. To achieve the purpose of distinguishing the homogeneous groups. The Pearson correlation analysis was performed by the Origin 2023 software (OriginLab Corporation, Northampton, MA, USA).

## 3. Results and Discussion

### 3.1. Effects of Different Additives on the Gel Properties of MP

#### 3.1.1. Gel Strength

**Figure 1:**
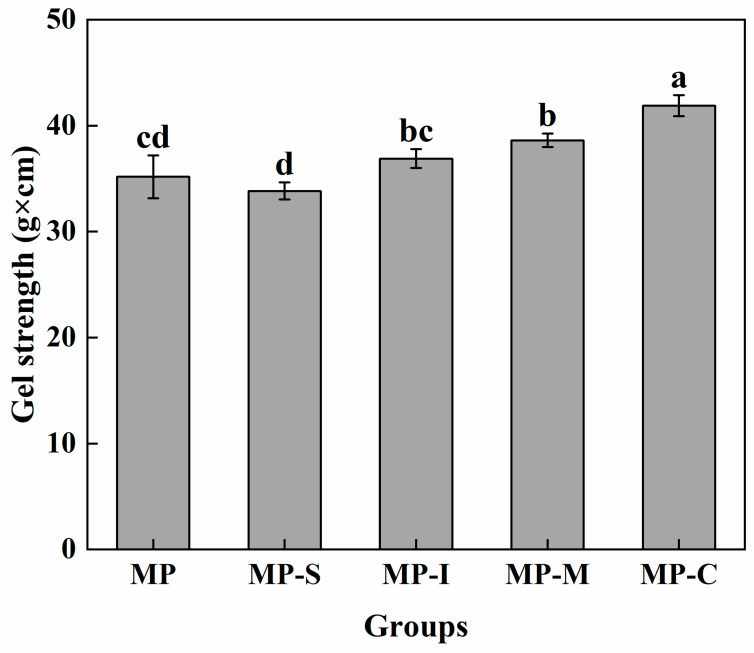
Effects of different additives on gel strength of myofibrillar gel. Note: MP: myofibrillar protein; MP-S: myofibrillar protein with 0.6% SPI; MP-I: myofibrillar protein with 0.6% inulin, MP-M: myofibrillar protein with 0.6% mixtures of SPI and inulin; MP-C: myofibrillar protein with 0.6% SPI–inulin conjugates, respectively. Different lowercase letters indicate a significant difference (*p* < 0.05).

Gel was assessed using a two-stage heating method (40 °C water bath for 30 min and 90 °C water bath for 20 min). The gel formed under this condition has a more compact and orderly network structure [27,28]. The gel strength of MP induced by different additives is depicted in Figure 1. The MP-S group exhibited the lowest gel strength at 33.842 ± 0.815 g·cm. These can be attributed to the compact spherical structure of SPI, limiting its interaction with MP under standard processing conditions and resulting in decreased gel strength [29]. Another study investigated the impact of natural SPI on the heat-induced gel of porcine myosin. It revealed that as the addition of SPI increased, the gel strength decreased. This phenomenon occurred due to the proteins of the two (SPI and porcine myosin) binding themselves and not interacting, resulting in a weaker gel strength. These findings align with the outcomes of the present study [11]. Additionally, conjugate with the MP-S group, inulin significantly enhanced the gel strength (*p* < 0.05). This enhancement was attributed to the hydrophilicity of inulin, allowing it to retain more moisture in the myofibrillar gel, therefore reinforcing the gel network and improving MP gel quality. The addition of the mixture also increased gel strength, possibly due to the synergistic effect of SPI and inulin [30]. Notably, the addition of the compound resulted in the highest gel strength at 41.897 g·cm, significantly (*p* < 0.05) surpassing other groups and exhibiting increases of 19.11%, 23.80%, 13.57%, and 8.50% compared to the first four groups, respectively. These could be attributed to the ultrasound-assisted Maillard reaction unfurling the SPI spherical structure, exposing its reaction groups, enhancing interaction with MP, filling the pores of the protein gel network more effectively, and forming an ordered structure of protein-polysaccharide protein [26,30,31].

#### 3.1.2. Gel Textural Properties

TPA is an easily quantifiable analytical technique that has been widely used in the food industry [32]. Hardness and elasticity represent the ability of food to resist and restore deformation, respectively. Adhesion is to describe the adhesion between food and food contact surface. Chewiness is the comprehensive embodiment of hardness, viscosity, and elasticity. Table 1 illustrates the effects of different additives on the hardness, springiness, gumminess, and chewiness of the myofibrillar gel. The hardness, springiness, gumminess, and chewiness of the MP-S group were the lowest, which were significantly lower than those of the MP group by 56.592 g, 0.026 g, 0.045 g, and 17.37 g, respectively. These results indicated that natural SPI could interfere with the formation of myofibrillar gel network. The main components of SPI, glycinin (11S) and β-conglycinin (7S), play a decisive role in gel properties. However, under normal processing conditions, these components do not exhibit structural changes, limiting their cross-linking with myofibril [18]. A lack of structural changes in 7S and 11S globulin under normal meat processing conditions hinders their interaction with meat proteins [19], leading to reduced functionality in MP gels [33]. The study suggests that 7S and 11S primarily impact the viscoelasticity and hardness of gels. The B subunits of 11S and β subunits of 7S form macromolecular aggregation through electrostatic interaction, significantly influencing gel properties [34]. Compared with the MP group, the MP-I and MP-M groups showed some improvement in hardness, springiness, gumminess, and chewiness, but the MP-C group exhibited the greatest improvement at 19.43%, 3.17%, 2.20%, and 26.40%, respectively. This improvement was attributed to the covalent combination of SPI with inulin, leading to increased solubility, expansion of the compact spherical structure, and filling of the structural pores in the myofibrillar gel network, ultimately enhancing the gel texture [35].

#### 3.1.3. WHC

**Figure 2:**
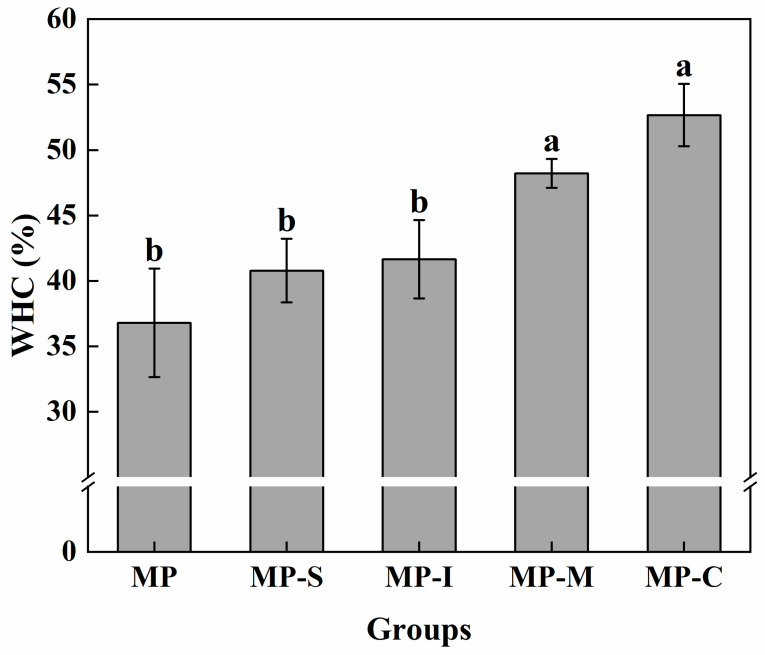
Effects of different additives on water retention of myofibrillar gel. Note: MP: myofibrillar protein; MP-S: myofibrillar protein with 0.6% SPI; MP-I: myofibrillar protein with 0.6% inulin, MP-M: myofibrillar protein with 0.6% mixtures of SPI and inulin; MP-C: myofibrillar protein with 0.6% SPI–inulin conjugates, respectively. Different lowercase letters indicate a significant difference (*p* < 0.05).

WHC retention reflects the binding capacity of the protein to water and represents the proportion of water contained in the gel network [26]. Figure 2 illustrates the effects of different additives on the water retention of the myofibrillar gel. Compared with the MP, the addition of SPI or inulin can improve the water retention of the gel. Moreover, the effect of adding SPI and inulin simultaneously was more significant. The self-gelatinization of SPI and the hydrophilicity of inulin contributed to trapping a significant amount of moisture in the myofibrillar gel, enhancing its water retention capacity [18,36]. Inulin has been previously shown to enhance the water retention capacity and gel strength of chicken myofibrillar gel [26]. This could be attributed to two primary reasons. First, the WHC of the gel was closely associated with its microstructure. The mixed gel with N-SPI exhibited an agglomerate structure, which was not conducive to the binding of proteins to water. Second, heating may cause the unfolding of natural proteins and the exposure of buried polar groups, therefore enhancing protein–protein interactions through hydrophobic binding to achieve the effect of gel enhancer [37,38]. Compared with other groups, the MP-C group had the best water-holding capacity of myofibrillar protein gel, which increased by 43.13%, indicating that the conjugate had a stronger water absorption capacity. The increase in water-holding capacity may be due to the increase of hydrogen bond content in the protein gel. After the ultrasound-assisted Maillard reaction, the polar groups of SPI were unfolded, and soybean globulin (11S) and β-conglycinin (7S) were involved in the process of myofibrillar protein gel, which made the myofibrillar protein gel have higher water-holding capacity [6,39].

#### 3.1.4. LF-NMR Proton Relaxation

**Figure 3:**
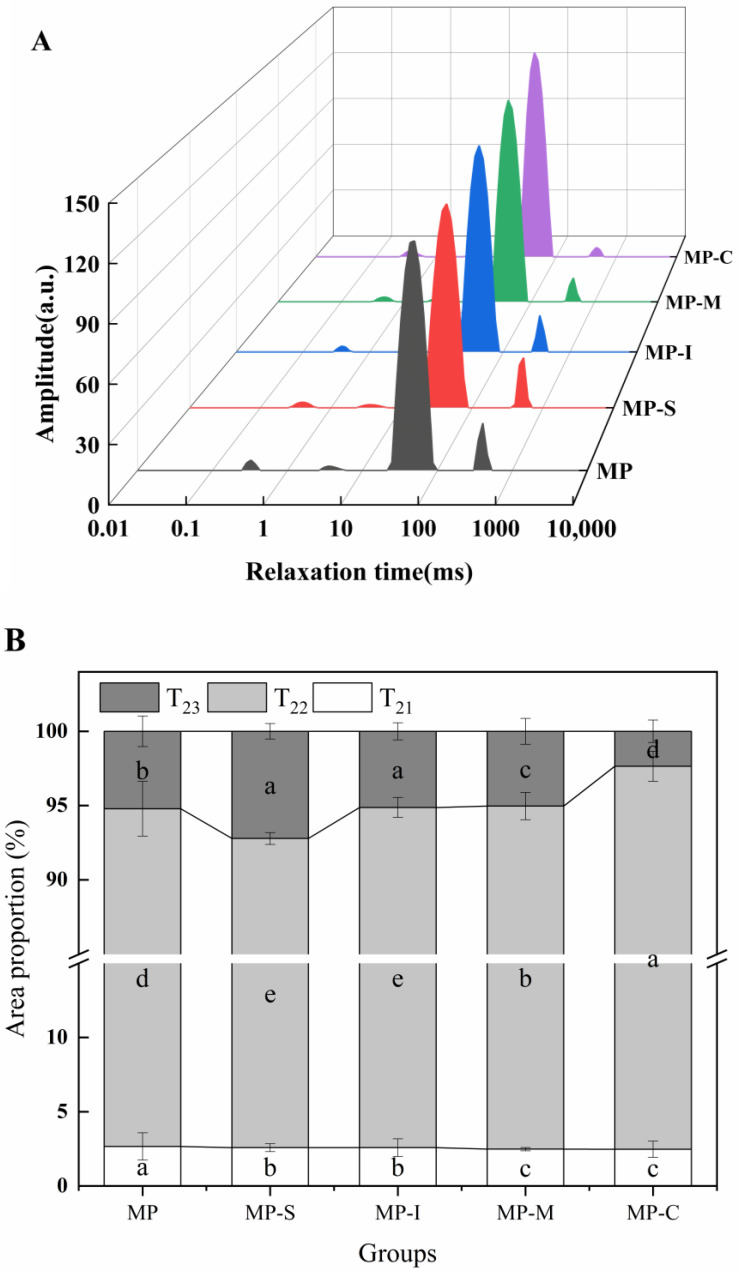
Effects of different additives on T_2_ relaxation time (**A**) and the peak area ratio (**B**) of myofibrillar gel. Note: MP: myofibrillar protein; MP-S: myofibrillar protein with 0.6% SPI; MP-I: myofibrillar protein with 0.6% inulin, MP-M: myofibrillar protein with 0.6% mixtures of SPI and inulin; MP-C: myofibrillar protein with 0.6% SPI–inulin conjugates, respectively. Different lowercase letters indicate a significant difference (*p* < 0.05).

LF-NMR technology rapidly detects specific information about water states in the protein gel system [40]. As depicted in Figure 3A, each group of samples exhibited four characteristic peaks. The relaxation time t_2_ within the 0–1 ms and 1–10 ms range represents water tightly bound to macromolecules, denoted as t_21_ and t_22_, respectively. The relaxation time t_2_ within the 10–100 ms range signifies water that was not easily mobile, referred to as t_23_. The relaxation time t_2_ within the 100–1000 ms range represents water outside the myofibrillar gel, labeled as t_24_ [41,42]. As shown in Figure 3B, compared with the control group, the area of p_21_ + p_22_ decreased, while the area of p_23_ increased in the MP-C treated group. This indicates that, at this stage, the protein gel structure formed could bind more water within the MP gel network. Moreover, free water molecules within the gel mechanism were immobilized [23]. The area of p_23_ in the MP-C treatment group significantly increased compared with that in the MP-S treatment group, suggesting that the combination of SPI and ultrasound improved protein solubility, reduced protein size, and increased the contact surface between protein and water [4]. Furthermore, ultrasound-induced structural changes in proteins may result in the exposure of more charged groups (NH_4_^+^, COO^−^) [43,44]. Consequently, during the heating process, these factors can enhance the formation of a more robust gel network structure in MPs. It was also observed that high-intensity ultrasound treatment could enhance the retention ability of low-salt gel for free water [45]. Therefore, following the addition of the conjugates, the content of t_23_ increased significantly. This suggests that the addition of the conjugate can elevate the content of non-mobile water (t_23_) within the myofibrillar gel, therefore restricting the fluidity of water in the gel network and trapping more water within the gel network (*p* < 0.05). The Maillard reaction induced structural changes in SPI, and the exposure of polar groups contributed to enhanced interaction with MP, forming a network structure that retained water molecules in the protein gel and reduced water loss [46], consistent with the gel strength results (Figure 1).

#### 3.1.5. Determination of Chemical Forces

**Figure 4:**
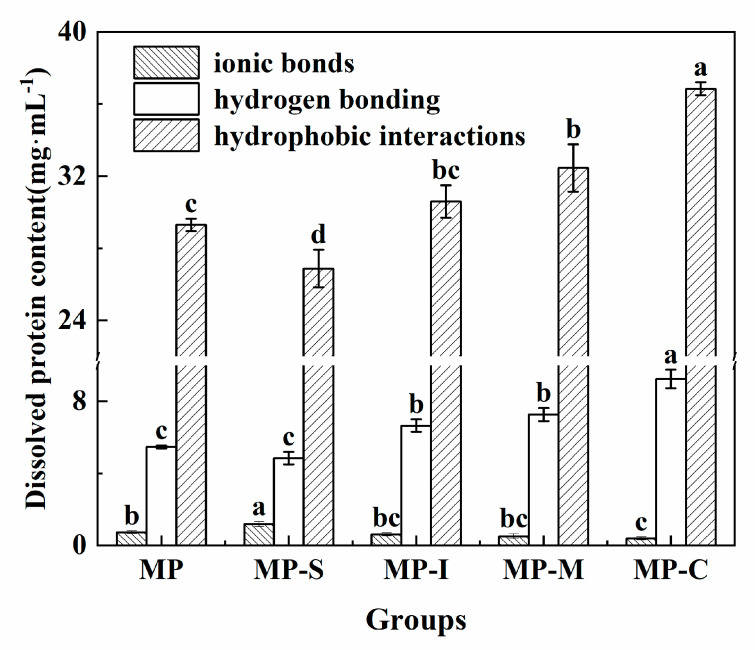
Effects of different additives on the chemical force of myofibrillar protein gel. Note: MP: myofibrillar protein; MP-S: myofibrillar protein with 0.6% SPI; MP-I: myofibrillar protein with 0.6% inulin, MP-M: myofibrillar protein with 0.6% mixtures of SPI and inulin; MP-C: myofibrillar protein with 0.6% SPI–inulin conjugates, respectively. Different lowercase letters indicate a significant difference (*p* < 0.05).

The role of intermolecular, including ionic bonds, hydrogen bonds, and hydrophobic interactions, was pivotal in the formation and maintenance of the three-dimensional network structure of myofibrillar gel [47]. Figure 4 illustrates the impact of different additives on the chemical forces of MP gel. Across all groups, hydrogen bonds and ionic bonds made minimal contributions to surimi gel structure, with hydrophobic interaction dominating the protein gel formation process. Compared with the MP group, the hydrogen bonding and hydrophobic interactions in the MP-C group were significantly increased by 69.07% and 25.77%, respectively [48]. The smallest proportion of ionic bonds resulted from the disruption of these bonds during the formation of the protein gel. Compared with the MP group, the hydrophobic interaction in the MP-C group exhibited a significant increase (*p* < 0.05). This increase was attributed to the introduction of hydrophilic groups after the covalent combination of SPI with inulin. These groups provided additional hydrogen donors, cross-linking with protein residues, therefore augmenting hydrophobic interaction [49]. Hydrophobic interaction played a crucial role in stabilizing SPI within the myofibrillar gel, contributing to the enhancement of G′ and structural properties [38]. It has been observed that exposure to hydrophobic domains was essential for the formation of myosin aggregates. The result of hydrophobic interaction was in a trend similar to that of the hydrogen bond index.

The hydrogen bond content demonstrated a certain correlation with WHC. Hydrogen bonding significantly influenced protein secondary structure content, particularly the relative content of β-sheet structure [50]. The regular transformation from α-helix to β-sheet during gelation necessitates protein rearrangement, leading to changes in hydrogen bonds and network structure [51]. Figure 4 indicates that the hydrogen bond content in the MP-C group was significantly higher than in other groups. This increase may be attributed to the exposure of polar groups after the Maillard reaction of SPI, promoting cross-linking with MP. The conjugate formed hydrogen bonds with amino groups exposed to MPs or with water molecules [49]. These findings align with the observed strength and WHC of the protein gel.

#### 3.1.6. Raman Spectroscopy

**Figure 5:**
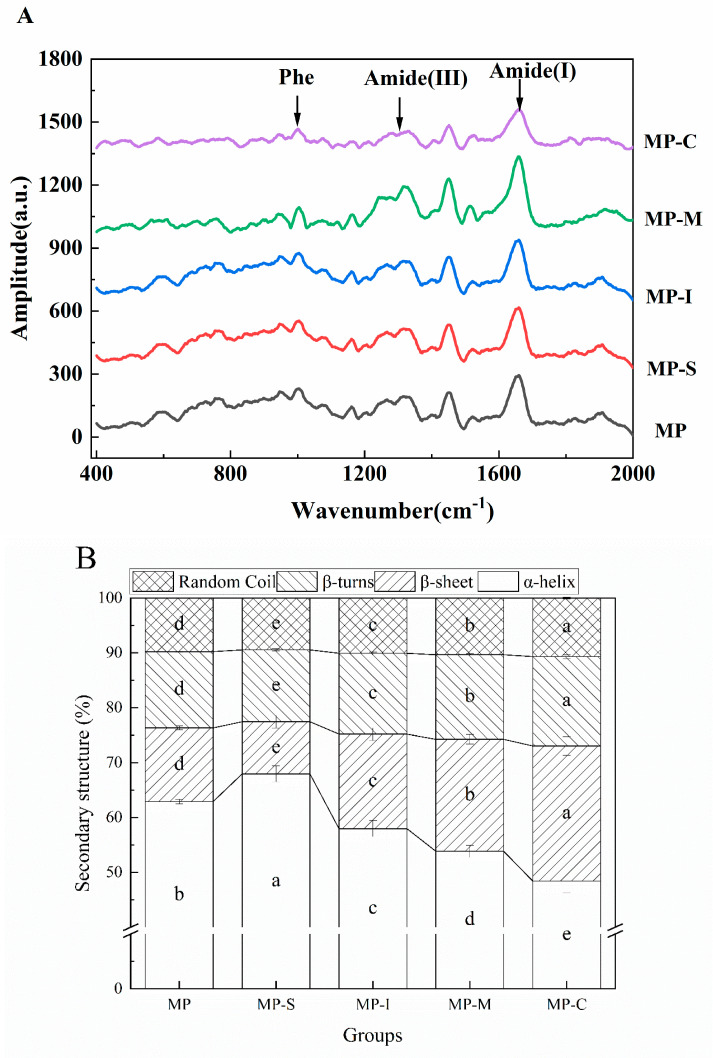
Effects of different additives on the Raman spectra (**A**) and protein secondary (**B**) structure content of myofibrillar protein gel. Note: MP: myofibrillar protein; MP-S: myofibrillar protein with 0.6% SPI; MP-I: myofibrillar protein with 0.6% inulin, MP-M: myofibrillar protein with 0.6% mixtures of SPI and inulin; MP-C: myofibrillar protein with 0.6% SPI–inulin conjugates, respectively. Different lowercase letters indicate a significant difference (*p* < 0.05).

The gel properties of proteins were correlated with changes in secondary structure content, making Raman spectroscopy a valuable tool for reflecting the secondary structure information of protein gels [52]. The most informative Raman bands for determining the secondary structure of proteins (α-helix, β-sheet, β-turn, random coil) include the amide I, amide II, and amide III bands. However, the Raman amide II band was often challenging to detect in proteins due to the small change in polarizability associated with amide II. Consequently, the present study primarily discusses the changes in the amide I and amide III bands [7]. Figure 5A illustrates the impact of different additives on the Raman spectra of myofibrillar gel. As observed in the figure, the amide I band (1600–1700 cm^−1^) and the amide III band (1200–1300 cm^−1^) serve as the main characteristic peaks of protein secondary structure, enabling the calculation of the content of protein secondary structure [7]. The contents of protein α-helix, β-sheet, β-turn, and random coiled structural units were calculated using the Alix formula, and the results are shown in Figure 5B.

As shown in Figure 5B, compared with the MP group, the α-helix content in the MP-S group significantly increased from 62.91% (*p* < 0.05) to 67.96%, while the β-sheet content decreased significantly from 13.40% (*p* < 0.05) to 9.47%. This shift may be attributed to the self-aggregation of SPI, which impedes the interaction between MPs, leading to higher α-helix content and lower β-sheet content. Except for the MP-S group, the amide I band in the Raman spectra of other surimi gels containing various additives shifted to a higher frequency. Quantitative analysis of the amide I band revealed a significant increase in the proportion of β-sheet and a decrease in the proportion of α-helix. However, compared with the MP group, the α-helix content of MP-C significantly decreased (*p* < 0.05) to 48.42%, and the β-sheet content significantly increased (*p* < 0.05) to 24.65%. This suggests that the α-helix structure of MP unfolded during the heat-induced gel process, exposing hydrophobic groups within the protein molecules. This enhancement of surface hydrophobicity in the protein promotes the transformation of α-helix to β-sheet in the protein gel. The increased β-sheet content suggested that ultrasonic treatment promoted more hydrogen bonds in protein gel formation, enhancing the interaction force between protein molecules, which was consistent with the gel strength [9,45,49]. In conclusion, the addition of the conjugate induced a significant conformational change in MP, promoting the transformation of α-helix to β-sheet and contributing to the later-stage formation of an ordered myofibrillar gel network.

#### 3.1.7. Viscoelasticity Measurements

**Figure 6:**
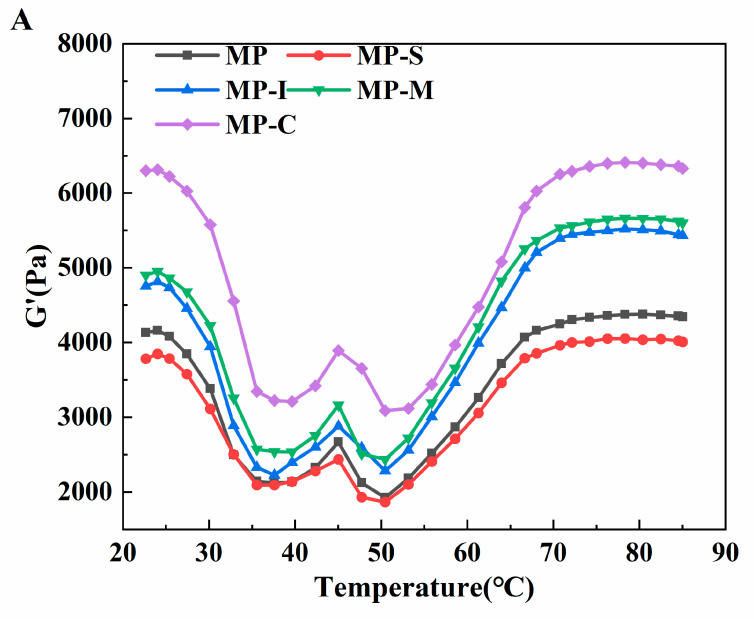

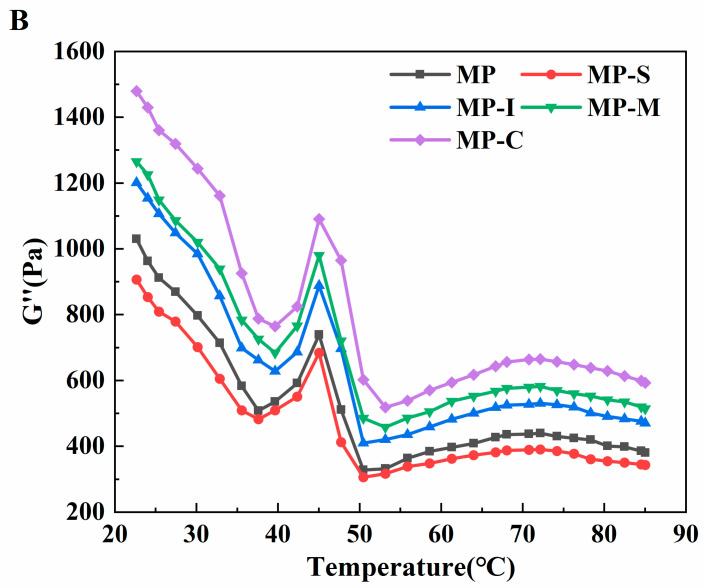
Effects of different additives on energy storage modulus (**A**) and loss modulus (**B**) of myofibrillar protein. Note: MP: myofibrillar protein; MP-S: myofibrillar protein with 0.6% SPI; MP-I: myofibrillar protein with 0.6% inulin, MP-M: myofibrillar protein with 0.6% mixtures of SPI and inulin; MP-C: myofibrillar protein with 0.6% SPI–inulin conjugates, respectively.

Dynamic rheological measurements involving storage modulus (G′) and loss modulus (G″) serve as valuable indicators for characterizing the formation and aggregation of protein gel networks. These parameters play a crucial role in evaluating the elasticity and viscosity of protein gels [53]. G′ represents the energy storage modulus, reflecting the elasticity of the protein gel network, while G″ represents the loss modulus, indicating the viscosity of the protein gel [54]. In Figure 6, the effects of different additives on the storage modulus and loss modulus of MP were depicted. Throughout the temperature-scanning process, the G′ and G″ curves of all samples exhibited a similar trend (Figure 6). The gradual increase of G′ and G″ from around 35 °C to approximately 45 °C suggests the unfolding of heavy myosin at low temperatures. These unfoldings allow for cross-linking between myosin filaments via hydrogen bonds, forming a weak protein network and enhancing the viscoelasticity of the sample [53,55]. The storage modulus peaks near 45℃, marking the myosin gelation stage. Between 45 °C and 50 °C, G′ gradually decreased, potentially indicating the spiral transformation of light myosin to irregularity. Partial disruption of hydrogen bonds due to heating could contribute to this decrease, disrupting already formed protein networks. This suggests a temporary destruction of the protein network structure [26]. Additionally, the degradation of the actin-myosin network structure is mediated by endogenous proteolytic enzymes, whose activity was optimal in the temperature range of 50–60 °C [56].

The gel strengthening stage initiates at 50 °C, leading to a significant increase in the G′ value with rising temperature. During this process, myofibril forms intermolecular covalent disulfide bonds undergoes cross-linking, and experiences protein side chain polymerization. These results in the formation of a thermally irreversible gel network structure, enhancing elasticity and hardness while stabilizing the development of the gel network [57]. Subsequently, both G′ and G″ decrease as the temperature approaches 90 °C, potentially due to the breakdown of hydrogen bonds at high temperatures. The final G′ values in descending order were MP-C > MP-M > MP-I > MP > MP-S. This indicates that the addition of the conjugate contributes to absorbing moisture overflowing from the protein gel through hydrogen bonds, distributing it evenly in the MP gel network, thus increasing the G′ value [26]. This observation aligns with the results obtained for protein gel strength.

As can be seen from Figure 6B, the trend of the G″ value mirrors that of G′. During the heating process, the G″ value gradually decreases within the range of 20–35 °C, with a peak value appearing near 45 °C. The value of G″ begins to increase when the temperature reaches 50 °C, indicating the onset of gelation or the formation of elastin networks. At 72 °C, it begins to decline. The final G″ value was much smaller than the G′ value, indicating that elasticity predominates during gelation [57].

### 3.2. Effect of Soybean Protein-Inulin Conjugates on the Conformation of MP of Spanish Mackerel

#### 3.2.1. Fluorescence Spectroscopy

Tryptophan, tyrosine, and phenylalanine, essential amino acids in protein molecules, were highly sensitive to changes in the microenvironment. Tryptophan, being widely present in various protein foods and dietary proteins, exhibits strong intrinsic fluorescence when excited at 295 nm. This property makes it a valuable tool for assessing changes in the tertiary structure of proteins [39].

**Figure 7:**
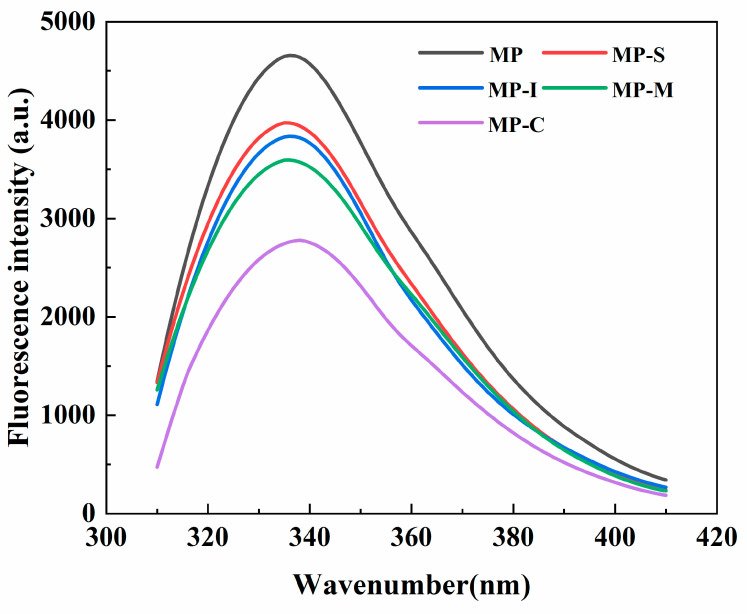
Effects of different additives on the endogenous fluorescence spectra of myofibrillar protein. Note: MP: myofibrillar protein; MP-S: myofibrillar protein with 0.6% SPI; MP-I: myofibrillar protein with 0.6% inulin, MP-M: myofibrillar protein with 0.6% mixtures of SPI and inulin; MP-C: myofibrillar protein with 0.6% SPI–inulin conjugates, respectively.

The endogenous fluorescence spectra of MP were measured to evaluate the influence of exogenous additions on protein conformation [58]. The effects of different additives on the endogenous fluorescence spectra of MP are presented in Figure 7. The addition of exogenous substances resulted in a reduction in fluorescence intensity. This decrease may be attributed to the unfolding of proteins and the exposure of tryptophan residues to the hydrophilic solvent environment. The interaction of exogenous substances with MP formed macromolecular complexes, leading to a decrease in tryptophan fluorescence intensity and fluorescence quenching [59]. Among the additives, the MP-C group exhibited the most significant decrease in fluorescence intensity, indicating that the addition of the conjugate promoted the development of the MP structure [26,60]. Additionally, the addition of the conjugate induced a redshift in the maximum fluorescence intensity of MP, signifying changes in the tryptophan residues’ microenvironment from non-polar to polar due to cross-linking and aggregation between proteins [61].

#### 3.2.2. Atomic Force Microscopy

In contrast to electron microscopy, AFM is a tool that probes complex food structures at the molecular level with minimal sample sizes. AFM’s imaging methods enable the assessment of molecular structures and interactions under near-natural conditions [47]. For instance, in the AFM image of the beet/pectin mixed sample, most of the molecules were protein-polysaccharide complexes, with proteins attached to one end of the pectin chain, explaining their excellent emulsification properties [62]. Previous studies utilized AFM to investigate the conformation of ovotransferrin and its complex with beet pectin, providing insights into the formation, binding, and dissociation of protein-polysaccharide systems [63].

**Figure 8:**
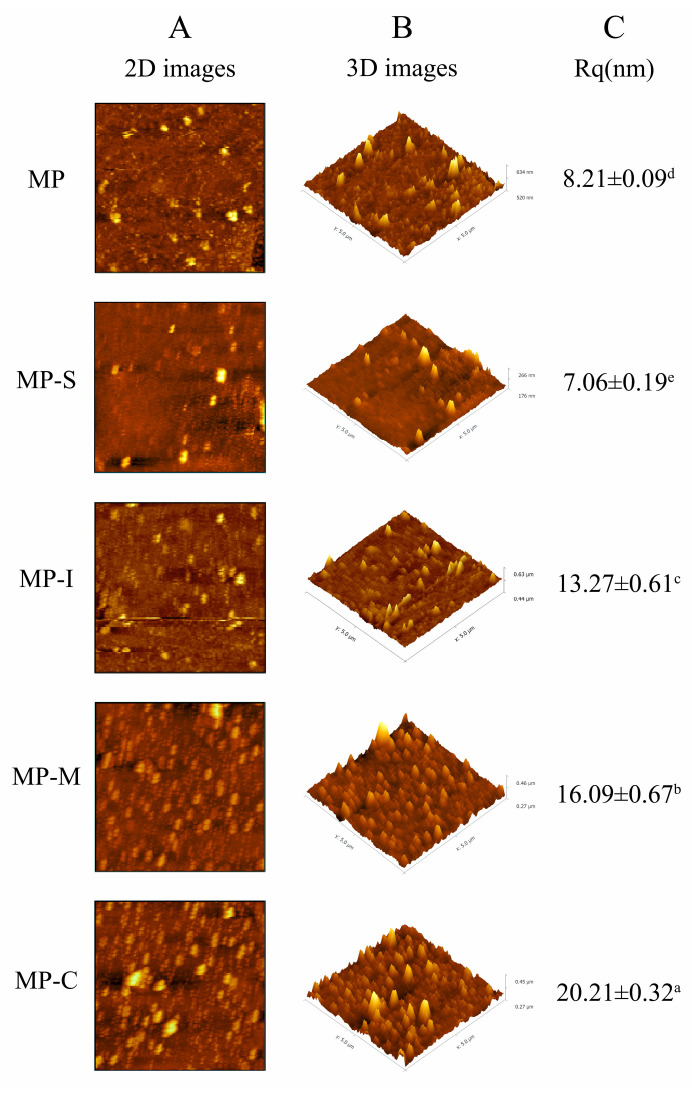
Effects of different additives on atomic force microscopy of myofibrillar protein. Note: MP: myofibrillar protein; MP-S: myofibrillar protein with 0.6% SPI; MP-I: myofibrillar protein with 0.6% inulin, MP-M: myofibrillar protein with 0.6% mixtures of SPI and inulin; MP-C: myofibrillar protein with 0.6% SPI–inulin conjugates, respectively. Different lowercase letters indicate a significant difference (*p* < 0.05).

AFM images offer high-resolution insights into the surface morphology of proteins. In the context of aquatic product processing, MP was a crucial focus due to its close association with rheological properties such as water retention, elasticity, and texture [27]. The microstructure of MP aggregates can be observed through AFM images. Figure 8 displays the effects of different additives on the atomic force microscopy of MP, including 2D and 3D images, along with the Rq value, representing the average roughness of the protein surface. From Figure 8, it was evident that the Rq value of the MP group was 8.09 nm. The addition of SPI significantly (*p* < 0.05) reduced it to 7.14 nm, indicating that SPI inhibited the aggregation of MP, possibly due to its self-gelatinicity hindering interaction with MP [18]. Except for the MP-S group, Rq values in the other groups increased, suggesting that the addition of various foreign substances can enhance the stability of MP. Additionally, it was observed that xylo-oligosaccharide inhibited the deterioration of functional properties and conformation by binding with myofibril, therefore improving the gel-forming ability of myofibril and increasing the stability of surimi myofibril [64]. The introduction of polysaccharide or starch to surimi protein was found to impact the microstructure of the heated surimi gel, resulting in increased hardness [65]. Following the addition of the conjugate, the Rq value significantly increased (*p* < 0.05) to 21.32 nm, indicating an enhanced degree of protein aggregation due to the binding of the conjugate with MP. This protein aggregation was a result of covalent cross-linking, contributing to the improved gelling ability and stability of MP. This outcome aligns with the observed increase in the gel strength of the protein [66].

### 3.3. Correlation Analysis of MP Gel Strength and Various Factors

**Figure 9:**
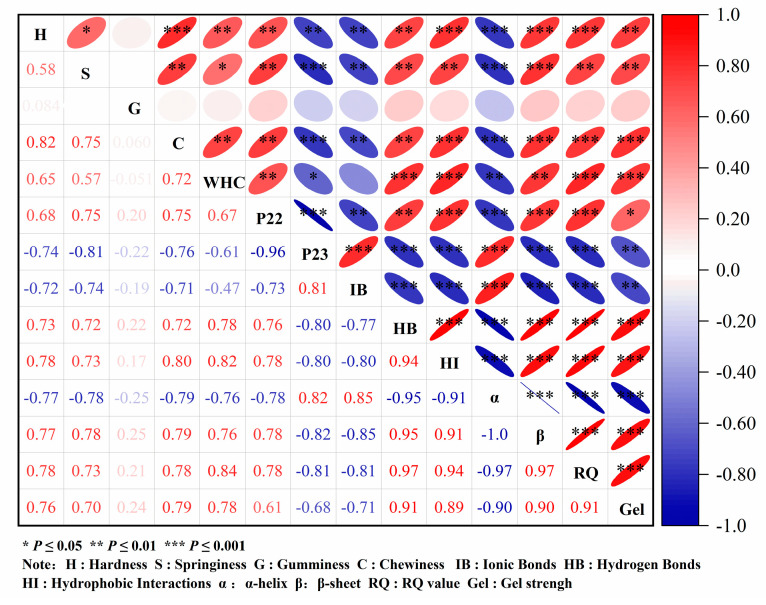
Correlation analysis of gel strength, texture, water distribution, water retention, chemical force, microstructure, and secondary structure parameters of myofibrillar protein gel.

A Pearson coefficient analysis was conducted to explore the relationship between MP gel strength and various factors such as texture, water distribution, water retention, chemical forces, secondary structure, and microstructure (Figure 9). Gel strength has a higher degree of positively correlated with hardness, elasticity, and chewability (*p* < 0.05), suggesting a positive effect on texture. Gel strength was positively correlated with P23 and water retention (*p* < 0.05) and negatively correlated with P24 (*p* < 0.05). Water retention was indicative of the binding ability of proteins to water, and P23 and P24 reflect the degree of binding between water and surrounding proteins in the gel network [58]. As illustrated in Figure 9, the stronger the binding ability between water and protein, the higher the degree of binding, and the more conducive it was to the formation of a gel network. Changes in the functional and structural properties of proteins influence alterations in their gel properties, consequently impacting the texture and sensory properties of surimi products [59]. In Figure 9, gel strength demonstrated a positive correlation with hydrogen bonds, hydrophobic interactions, and β-sheet content (*p* < 0.05) and a negative correlation with ionic bonds and α-helix content (*p* < 0.05). The study also examined the relationship between texture characteristics and characteristic bands of Raman spectra, estimating Raman spectra through principal component analysis [67]. Texture properties exhibited positive correlations with the proportions of β-sheet, β-turns, and random coil while showing negative correlations with the proportions of α-helix. These findings align with the results of this study [67]. Moreover, it was observed that moderate oxidation-induced α-helix development could increase the hydrophobic and covalent bond content of the protein surface, promote the cross-linking aggregation of MP, and subsequently enhance the gel strength of the protein [59]. The microstructure reflects the density of the three-dimensional network of the protein gel. As depicted in Figure 9, there was a significant (*p* < 0.05) positive correlation between gel strength and microstructure. The higher the gel strength of the protein, the denser the microstructure, and the microstructure of surimi was found to be related to gel strength [68]. In conclusion, changes in the secondary structure, interactions, microstructure, and water state of MP gel were crucial factors influencing the texture and strength of the protein gel.

## 4. Conclusions

The SPI–inulin conjugate significantly enhances the gel strength, texture, water retention, and thermal stability of the protein gel. At the same addition amount, the compound reaches maximum gel strength and water retention, measuring 41.897 g·cm and 52.67%, respectively. Combined with rheological analysis, it was concluded that the addition of conjugate can increase the G′ value of protein gel, and the springiness of protein occupies the main part during the gelation process. This increase in water retention was attributed to the elevated proportion of water content that was not easily flowing, with water molecules being covered inside the protein gel, resulting in reduced water loss. Correlation analysis results demonstrate a positive correlation between gel strength and texture, WHC, the proportion of immobile water, hydrophobic interaction, hydrogen bond, and α-helix content (*p* < 0.05). Through chemical force, AFM, fluorescence analysis of conjugates can effectively improve the conjugate characteristics of the reason. The addition of the conjugates promoted the unfolding of the protein and the exposure of the tryptophan residues to the hydrophilic solvent environment, resulting in the transformation of the α-helix to the β-sheet in the protein gel. Because of its self-gelatinization, SPI hinders the interaction of MP and inhibits the aggregation of MP. The conjugates can bind to MP, enhance the aggregation of proteins, and enhance the formation of protein gel network structure. This is the mechanism by which the SPI–inulin conjugate affects the gel properties and molecular conformation of MP. Therefore, the addition of the conjugate effectively improves the network structure of the protein gel, providing theoretical support for surimi processing technology.

## Figures and Tables

**Table 1:** Effects of different additives on the texture of myofibrillar protein gel.

Groups	Hardness (g)	Springiness	Gumminess	Chewiness (g)
MP	627.081 ± 12.273 ^bc^	0.916 ± 0.015 ^ab^	0.726 ± 0.084 ^a^	392.770 ± 18.868 ^b^
MP-S	570.489 ± 22.885 ^c^	0.890 ± 0.012 ^b^	0.681 ± 0.049 ^a^	375.400 ± 19.489 ^b^
MP-I	648.890 ± 12.263 ^bc^	0.919 ± 0.013 ^ab^	0.729 ± 0.072 ^a^	420.275 ± 26.135 ^b^
MP-M	677.893 ± 42.694 ^ab^	0.924 ± 0.007 ^a^	0.730 ± 0.081 ^a^	429.929 ± 26.317 ^b^
MP-C	748.947 ± 65.197 ^a^	0.945 ± 0.013 ^a^	0.742 ± 0.058 ^a^	496.459 ± 37.582 ^a^

Note: MP: myofibrillar protein; MP-S: myofibrillar protein with 0.6% SPI; MP-I: myofibrillar protein with 0.6% inulin, MP-M: myofibrillar protein with 0.6% mixtures of SPI and inulin; MP-C: myofibrillar protein with 0.6% SPI–inulin conjugates, respectively. Different lowercase letters indicate a significant difference (*p* < 0.05).

## Data Availability

The original contributions presented in the study are included in the article, further inquiries can be directed to the corresponding author.
